# Designing and expression of novel recombinant fusion protein for efficient antigen screening of SARS-CoV-2

**DOI:** 10.1186/s13568-024-01719-y

**Published:** 2024-07-11

**Authors:** G. Vinaya Chandu Vidyasagar, P. V. Janardhan Reddy, M. Md. Ghouse, T. C. Venkateswarulu, P. B. Kavi Kishor, Prashanth Suravajhala, Rathnagiri Polavarapu

**Affiliations:** 1 Genomix CARL Pvt. Ltd, YSR Kadapa, Pulivendula, 516 390 Andhra Pradesh India; 2 Amrita School of Biotechnology, Amrita Viswa Vidyapeetham, Clappana, 690525 Kerala India; 3 Department of Biotechnology, Vignan’s Foundation for Science, Technology & Research, Vadlamudi, Guntur, 522 213 India; 4 Department of Genetics, Osmania University, Hyderabad, 500 007 India

**Keywords:** COVID-19, SARS-CoV-2, Variants, Rapid antigen assay, Variants of concern, Variants of importance

## Abstract

Corona virus disease 2019 (COVID-19) pandemic caused by severe acute respiratory syndrome coronavirus 2 (SARS-CoV-2), claimed millions globally. After the report of the first incidence of the virus, variants emerged with each posing a unique threat than its predecessors. Though many advanced diagnostic assays like real-time PCR are available for screening of SARS-CoV-2, their applications are being hindered because of accessibility and cost. With the advent of rapid assays for antigenic screening of SARS-CoV-2 made diagnostics far easy as the assays are rapid, cost-effective and can be used at point-of-care settings. In the present study, a fusion construct was made utilising highly immunogenic B cell epitopes from the three important structural proteins of SARS-CoV-2. The protein was expressed; purified capture mAbs generated and rapid antigen assay was developed. Eight hundred and forty nasopharyngeal swab samples were screened for the evaluation of the developed assay which showed 37.14% positivity, 96.51% and 100% sensitivity and specificity respectively. The assay developed was supposed to identify SARS-CoV-2 wild-type as well as variants of concern and variants of importance in real-time conditions.

## Introduction

COVID-19 pandemic caused by the SARS-CoV-2 affected 772 million populations and claimed around 7 million lives worldwide (WHO, 2023). Presently only symptomatic therapy is available for COVID-19 despite the accessibility of various vaccines. SARS-CoV-2 still co-exists with the human population, thus emphasising the importance of new diagnostic methodologies for effective and accurate diagnosis of the COVID-19 (Yadegari et al. 2023). SARS-CoV-2 is an enveloped virus with single stranded 29.9 kb RNA genome with four structural proteins namely surface glycoproteins or spike (S), nucleocapsid (N), envelope (E) and membrane (M) (Wu et al. 2020). Of these, S is more prone to mutations (Farhud and Mojahed 2022) while N is the least prone to mutations (Naqvi et al. 2020; Che et al. 2004). Utilisation of diagnostic assays played a major role in control management of COVID-19. Though the gold standard real-time RT-PCR has proved its ability in effective screening of the SARS-CoV-2, it is having its own limitations like long turnaround time because of sample load, cost effectiveness, needs skilled personnel and centralised specialised laboratory facilities (Dong et al. 2023). The advent of rapid diagnostic assays made it easy for faster diagnosis of COVID-19 at a lower cost, in low-income countries as well as screening of mass population in very short intervals of time with minimal technical expertise (Baldanti et al. 2022).

With the evolution of new mutants and variants of concern (VoC) like Delta, Omicron along with its sub variants and the new JN.1, the COVID-19 disease is still a threat to human kind. While the screening of the mutants and VoCs is difficult with the existing point-of-care rapid antigen assays with majority of targeting N protein (Fujisawa et al. 2023; He et al. 2004) several mutant forms of spike protein evade the immune response (Chen et al. 2020; Harvey et al. 2021). Reports also identified a mutation in the N region that decreases the sensitivity up to 1000-fold (Bourassa et al. 2021; Jian et al. 2022). The present study focuses on the development of rapid antigen assays for screening SARS-CoV-2 which are more sensitive and can even diagnose mutants if any.

## Materials and methods

### Design of the construct

To design the recombinant construct, the gene sequences of the surface glycoprotein (S), membrane (M) and nucleocapsid (N) were taken from the SARS-CoV-2 genome reference sequence Wuhan-Hu-1 (Accession Number MN908947) from the National Centre for Biotechnology Information (NCBI) database. The fragments were selected in such a way that they will not fall in the region of mutation or not in VoCs like Delta, Omicron (B.1.1.529) or variants of interest (VoI) like Omicron (XBB. 1.5; XBB.1.16; B.A. 2.86) (Farhud and Mojahed 2022; Cosar et al. 2022). The B cell epitopes of the three genes were identified using IEDB (Jespersen et al. 2017) and from the theoretical studies reported by Grifoni et al. (2020). The sequences deduced were joined with flexible linker GGGGS which allows interaction between domains (Chen et al. 2013) to get a functional fusion protein after adding the start and stop codons at both 5′ and 3′ ends respectively. The constructed fusion protein sequence was reverse translated to get the nucleotide sequence. The constructed protein’s secondary structure was determined by the Prabi server at SOPMA (Obaidullah et al. 2021). The amino acid composition, physicochemical properties of the constructed protein, including molecular weight, isoelectric point, net charge at pH7, half-life in the mammalian reticulocytes, and instability index, were estimated using Protparam (Gasteiger et al. 2005). The 3D structure of the chimeric protein was generated using the I-Tasser server (Zheng et al. 2021; Yang and Zhang 2015) and viewed on VMD Molecular Graphis Viewer (Humphrey et al. 1996). Antigenicity of the fusion protein was evaluated using VaxiJen (Nosrati et al. 2019).

### Transformation and expression

The recombinant fusion gene was synthesized commercially in pET28a (+) vector (Gene Universal, USA) for expression. The recombinant fusion plasmid was transformed into *E. coli* BL21 cells using calcium chloride chemical method (Sambrook and Russell 2001) on Luria-Bertani (LB) agar plates with kanamycin as selection marker. The colonies formed were checked for the presence of plasmid using alkaline-lysis method and further by polymerase chain reaction (PCR) using primers for T7 promoter and T7 terminator. The positive fusion clones were expressed using 1 mM IPTG and the recombinant fusion protein was separated on the affinity column using Ni-NTA resin (in-house Genomix) under denaturing conditions. The quality of the expressed fusion protein was checked on 12% SDS-PAGE gels, while the concentration of the purified recombinant protein was determined using nanodrop spectrophotometer (Thermo Scientific).

### Development of rapid antigen assay

**Fig. 1:**
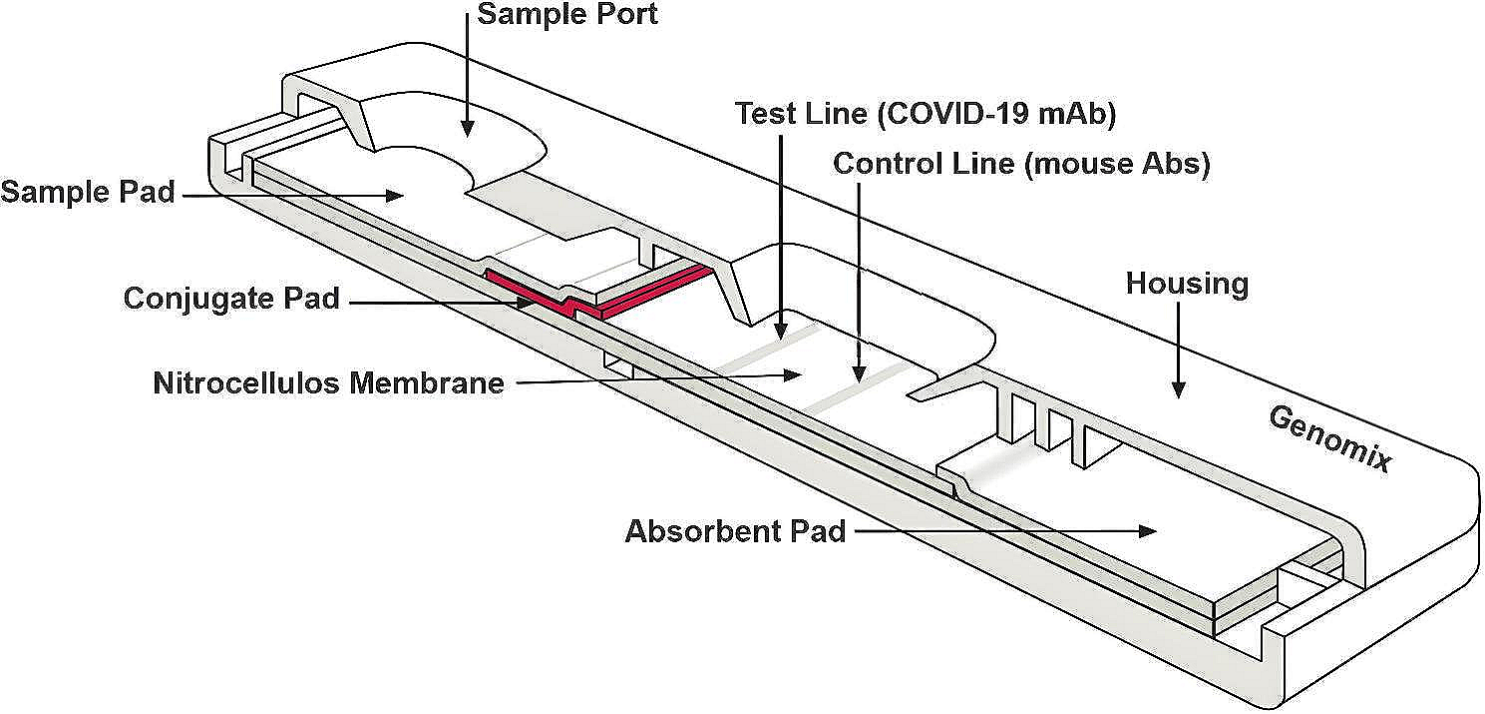
Image showing the layout of the rapid antigen test cassette. The strip in the cassette housing comprises of a sample pad and a conjugate pad with colloidal gold conjugated mouse polyclonal antibodies followed by the nitrocellulose membrane coated with mAbs against fusion antigen at T and goat anti-mouse antibodies at C position. The membrane is overlayed by the absorbent pad

The recombinant fusion protein was used for the development of monoclonal antibodies which were synthesized commercially (Dx Sys Inc., USA). The polyclonal antibodies were raised in mice (in-house) and IgG antibody was purified on protein A column and was tagged with colloidal nano gold (in-house Genomix). The test line (T) on the nitrocellulose membrane (MDI, India) was coated with 1 mg/ml of the monoclonal antibodies while the control line (C) was coated with 1 mg/ml of goat anti-mouse antibodies (in-house). The conjugate pad consists of colloidal gold conjugated polyclonal mouse antibodies (Fig. 1). The test strip is to be supplied with a proprietary lysis buffer for the usage of the assay.

If SARS-CoV-2 antigen is present in the sample, it binds to the colloidal nano gold conjugated mouse polyclonal antibodies on the conjugate pad. By capillary action, the complex moves towards the absorbent pad. The SARS-CoV-2 antigen in the complex binds to the mAbs on the test line, thus gives the purple line at T. If no antigen is present in the sample, no line appears at T while the unbound colloidal gold conjugated.

polyclonal antibodies bind with goat anti-mouse antibodies on the control line, thus gives the purple line at C.

### Sample collection and usage

A total of 840 parallel nasopharyngeal or oropharyngeal swab samples were collected from the subjects attending a tertiary care hospital with suspicion of COVID-19 infection along with a sample in VTM for real-time RT-PCR with informed consent (Vidyasagar et al. 2023). The swabs were inserted immediately in the lysis buffer vial and mixed well and loaded onto the sample well of the test cassette and waited till the colour line appeared either on T and/or C lines. No results were read after 20 min of adding the sample.

### Performance evaluation of the developed assay

The developed assay was evaluated against the standard real time RT-PCR for screening of SARS-CoV-2 as per manufacturer’s instructions (Huwel, India). Sensitivity and specificity were calculated for the assays performed on samples collected and screened as per earlier studies (Vidyasagar et al. 2023).

### Limit of detection (LOD) determination

The analytical performance of the developed rapid antigen assay was assessed by testing the LOD with different concentrations of recombinant SARS-CoV-2 recombinant fusion protein diluted in phosphate buffered saline (pH 7.4) as described (Fischl et al. 2024; Weishampel et al. 2022). The LOD was confirmed as the lowest concentration of recombinant protein that was detected ≥ 95% of the time and all the rapid assays were read with in 20 min.

## Results

### Recombinant fusion construct

**Fig. 2:**
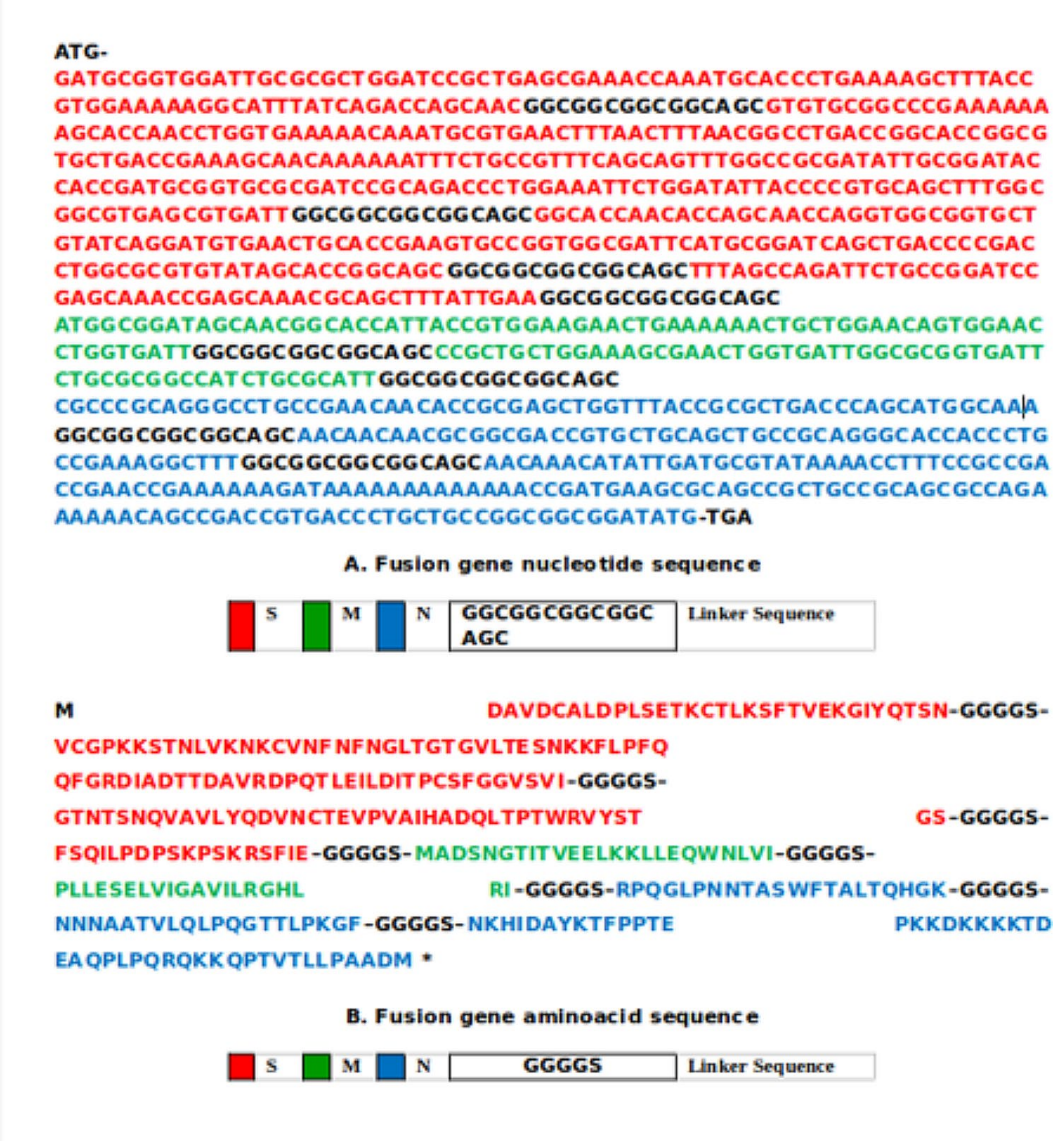
Nucleotide and amino acid sequences of fusion gene of SARS-CoV-2. (**A**) Nucleotide sequence of the recombinant fusion gene with selected reverse translated sequences from Spike, membrane and nucleocapsid genes of SARS-CoV-2 which are joined the linker sequence. (**B**) Aminoacid sequence of the fusion protein with selected epitopes from spike, membrane and nucleocapsid proteins of SARS-CoV-2 which are joined by the flexible linker sequence GGGGS. Start and stop codons were added at 5’ and 3’ ends respectively

**Fig. 3:**
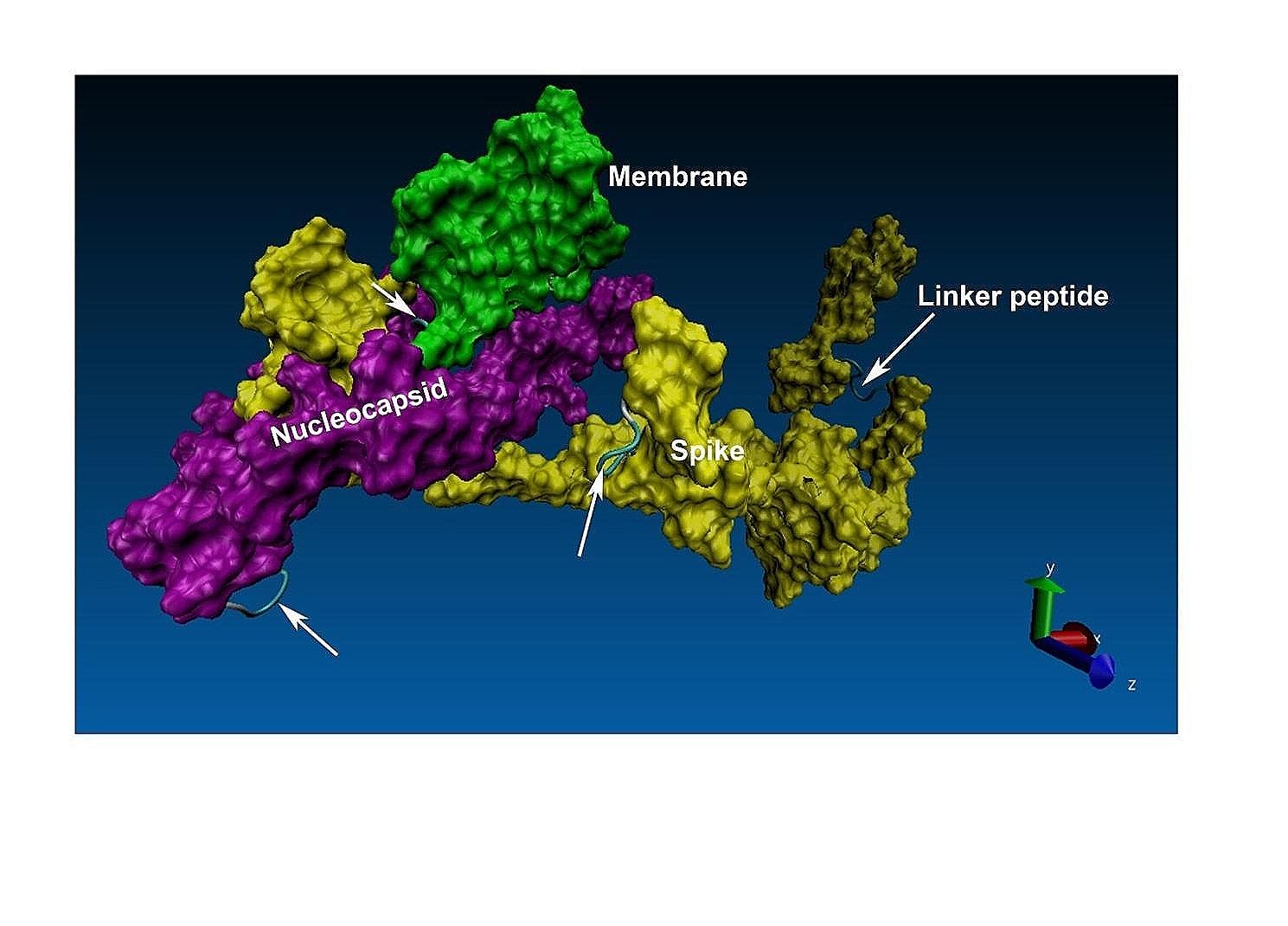
3D structure of the fusion protein. The selected epitopes of spike (yellow), membrane (green) and nucleocapsid (purple) in the recombinant fusion protein. Arrows represent the flexible GGGGS linkers for joining the selected epitopes to form a functional protein

The recombinant fusion construct is of 1014 bp which includes five epitopes from S protein, 2 from M protein and 3 from N protein (Table 1) from which the complete fusion gene and protein sequence was designed (Fig. 2). The Protparam analysis of the synthetic fusion protein revealed 337 amino acids with a molecular weight of 35.16 kDa and a theoretical isoelectric point of 8.64. The estimated half-life of the fusion protein in human reticulocytes is calculated as 30 h. With the instability index calculated at 44.78, the protein is unstable. The predicted aliphatic index of the fusion protein is 72.88 and the calculated Grand Average of Hydropathicity (GRAVY) is -0.381. The SOPMA result for the fusion protein revealed 16.91% alpha helix (57 AAs), 25.52% extended strand (86 AAs), 10.68% beta turns (36 AAs) and 46.88% random coil (158 AAs). Overall protective antigen prediction is predicted as 0.5491, indicating that the fusion protein is a probable antigen. The I-Tasser generated 3D image is shown in the Fig. 3.

**Table 1:** Table showing the epitope sequences from different structural proteins of SARS-CoV-2 used in the construction of fusion protein

Protein	Epitope sequence	Start	End
**Spike (S)**	DAVDCALDPLSETKCTLKSFTVEKGIYQTSN	287	317
	VCGPKKSTNLVKNKCVNFNFNGLTGTGVLTESNKKFLPFQQFGRDIADTTDAVRDPQTLEILDITPCSFGGVSVI	524	598
	GTNTSNQVAVLYQDVNCTEVPVAIHADQLTPTWRVYSTGS	601	640
	FSQILPDPSKPSKRSFIE	802	819
	FGAGAALQIPFAMQMAYRFNGI	888	909
**Membrane (M)**	MADSNGTITVEELKKLLEQWNLVI	1	24
	PLLESELVIGAVILRGHLRI	132	151
**Nucleocapsid (N)**	RPQGLPNNTASWFTALTQHGK	41	61
	NNNAATVLQLPQGTTLPKGF	152	171
	NKHIDAYKTFPPTEPKKDKKKKTDEAQPLPQRQKKQPTVTLLPAADM	354	400

### Confirmation of recombinant fusion clone

**Fig. 4:**
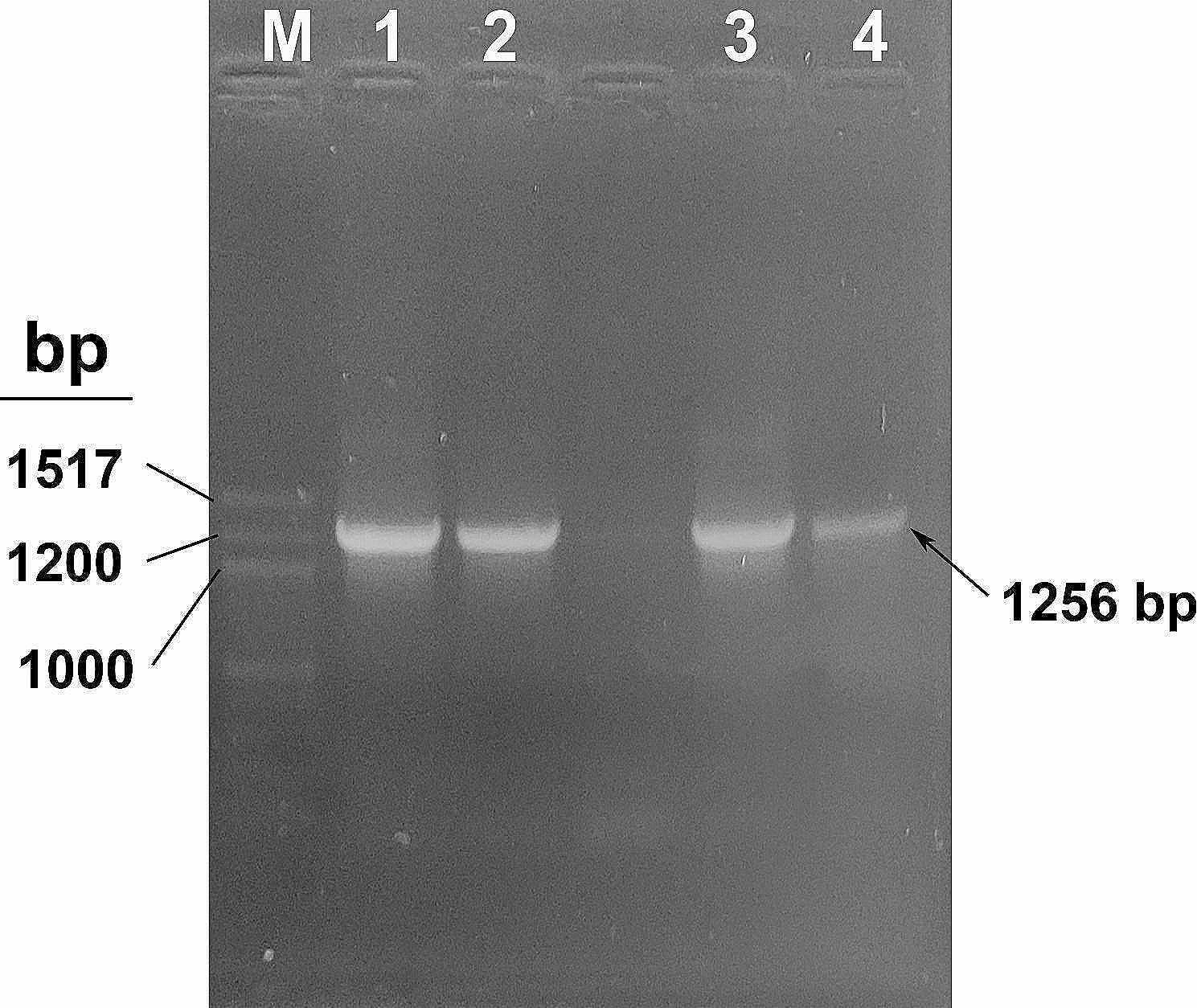
Agarose gel showing the amplification of the fusion gene. PCR performed using T7 promoter and T7 terminator universal primers shows the 1256 bp amplified fusion plasmid cloned in pET28a(+) vector (lanes 1–4) run against the 100 bp ladder (lane M) (NEB)

The PCR performed using T7 universal primers to confirm the presence of the recombinant fusion gene in the pET28a(+) plasmid revealed a 1256 bp fragment (Fig. 4) on 1% agarose-TAE gel.

### Expression and purification of the fusion protein

**Fig. 5:**
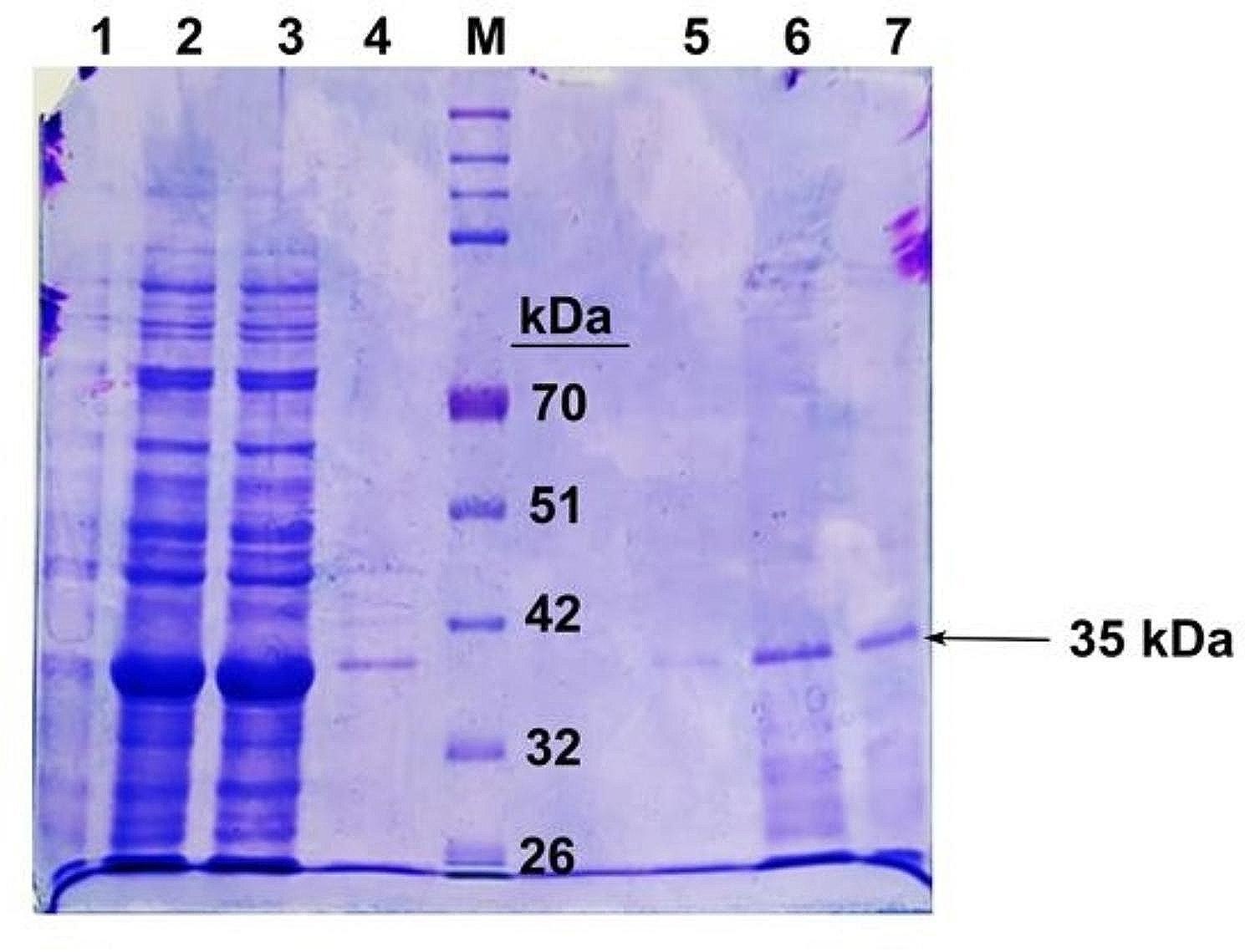
SDS-PAGE gel showing the purified recombinant fusion protein.  Lane 1 showing the pre-induction of the protein where no expression is seen; Lanes 2–3 showing the induction of recombinant fusion protein induced with 1mM IPTG; Lanes 4–7 showing the 35 kDa eluted purified fusion protein. Lane M showing the pre-stained broad range protein marker (Puregene)

 The desired 35 kDa recombinant fusion protein expression was evaluated on 12% SDS-PAGE (Fig. 5) with N terminal 6x His-tag. The concentration of the purified recombinant protein determined using nanodrop spectrophotometer was 1 mg/ml.

### Development of antigen rapid assay

**Fig. 6:**
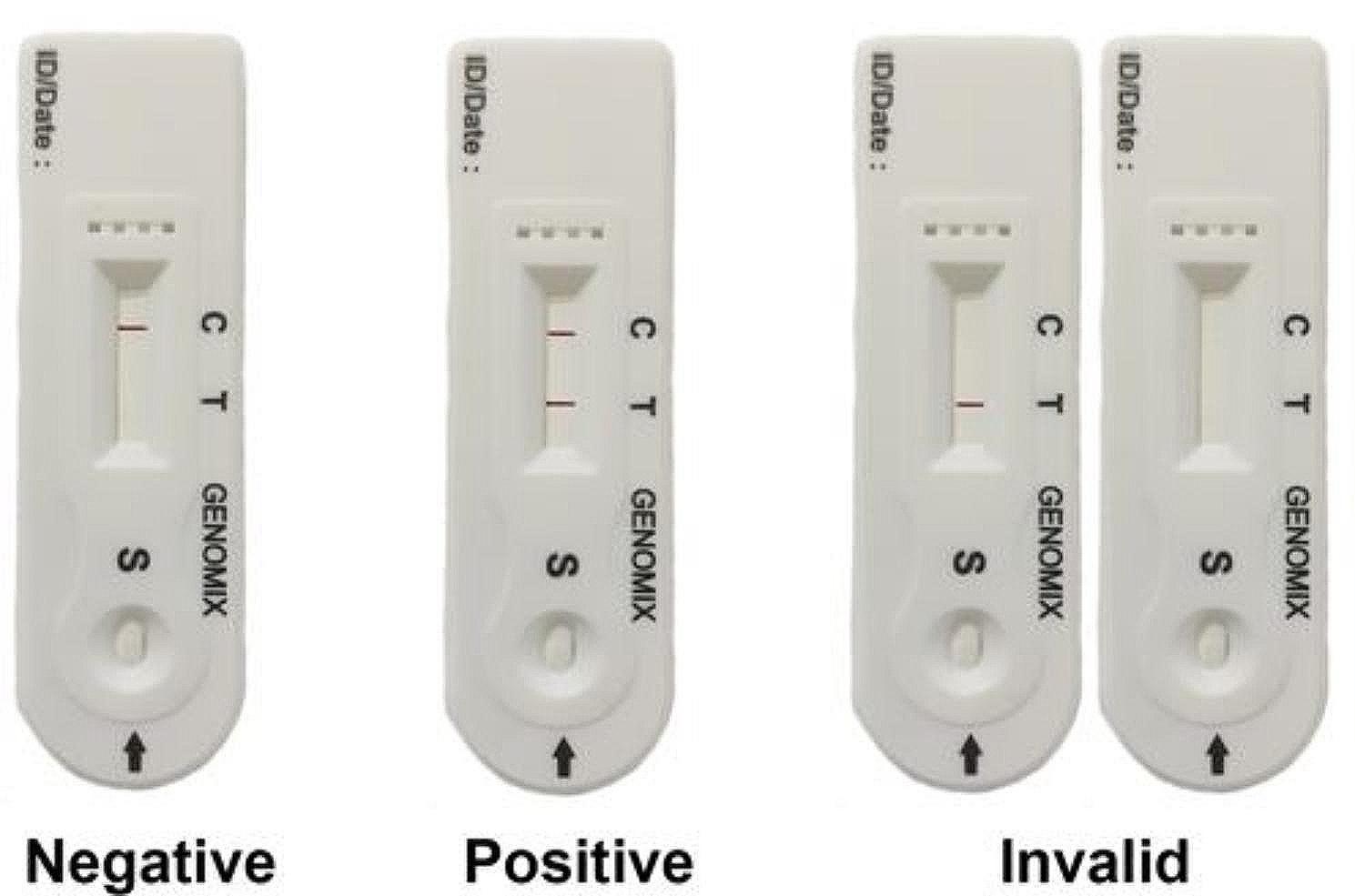
Image showing the reading patterns of the rapid antigen assay.  If purple line appears only at C position, the sample is negative for SARS-CoV-2. If purple lines appear both at C and T positions, the sample is positive for SARS-CoV-2 irrespective of the thickness of line at T. If no purple line appears at C with /without line at T position, the test is invalid and needs repetition with a new test device or sample

**Fig. 7:**
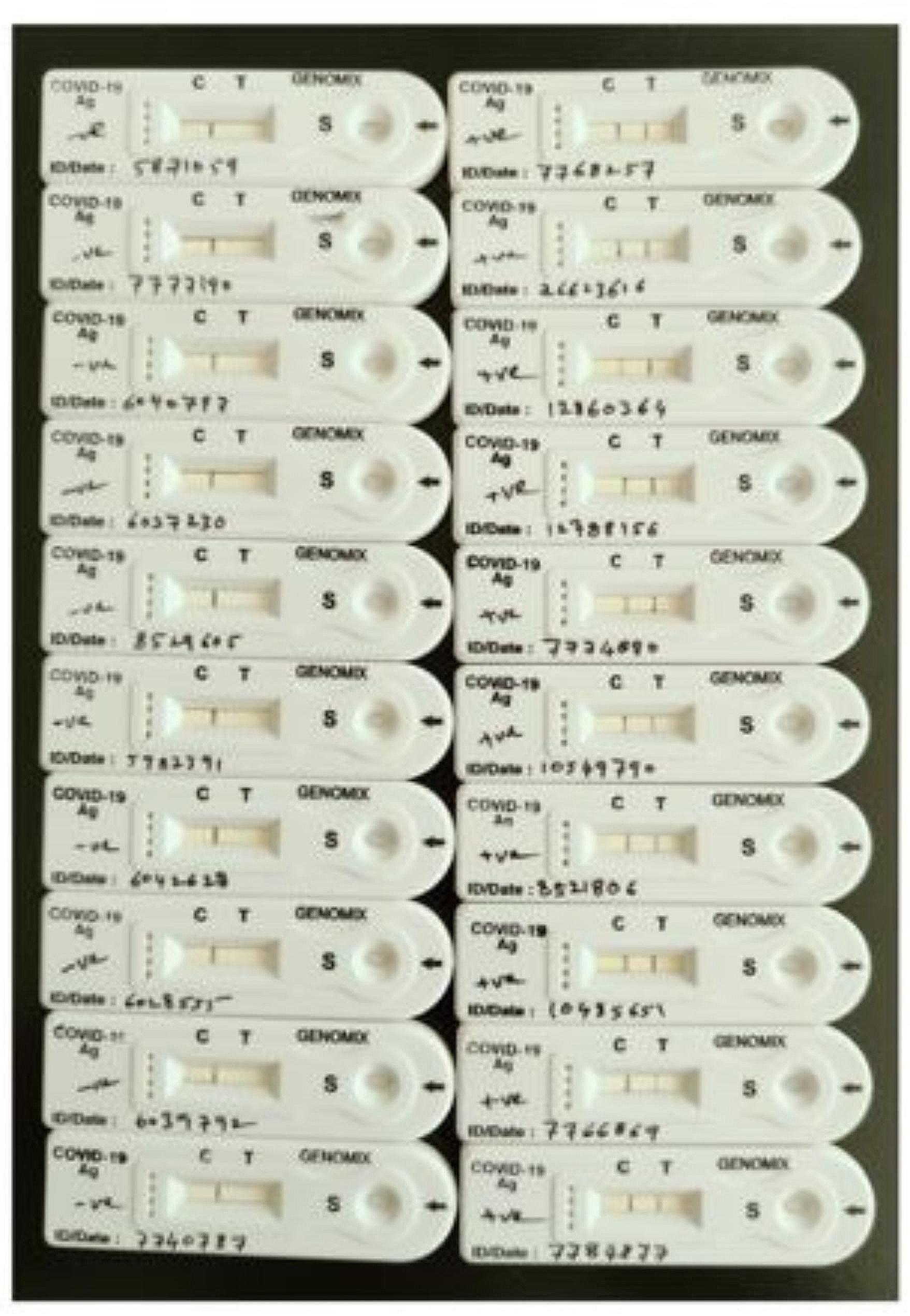
Results of rapid antigen assay using nasopharyngeal swab samples. If purple line appears at C position, then the samples are negative for SARS-CoV-2 (shown on left side of the image) and if purple line appears both at C & T positions, then the samples are positive for the presence of SARS-CoV-2 (shown on right side of the image)

From the 840 samples collected, a total of 315 samples were found positive for SARS-CoV-2 by real time RT-PCR while 525 samples were found negative. The same samples were used for antigen screening by the in-house developed rapid assay which resulted in 312 positives and 528 negatives with an overall positivity of 37.14%. The reading of the results was carried out as shown in Fig. 6. The representative images were shown in the Fig. 7.

### Performance of the developed antigen assay

The antigen rapid assay developed was found to be at par with the real-time RT-PCR evaluated. The assay showed a sensitivity of 96.51%, a specificity of 100% and accuracy of 98.69% at 95% CI (Table 2).

**Table 2:** Table showing the performance evaluation of the antigen rapid test against the real time RT-PCR for screening SARS-CoV-2

Rapid antigen assay	Real time RT-PCR				
	Positive	*n*	Negative	*n*	Total
Positive	True positive	304	False positive	0	304
Negative	False negative	11	True negative	525	536
Total		315		525	840

### Analytical sensitivity

The detection ability of the recombinant fusion protein was tested using the quantified purified fusion protein. The LOD for the recombinant protein was determined to be 0.327 ng/ml.

## Discussion

To the authors’ knowledge, this is the first-time structural information on multiple viral proteins has been used to generate a native immunogen, whose mAbs capture viral proteins. Rapid antigen assays are very important for tracking the spread of an infectious disease during a pandemic. Though the rapid assays for SARS-CoV-2 offer benefits over real-time RT-PCR assays but lag behind in terms of sensitivity (Funabashi et al. 2021). Most of the rapid antigen assays were designed using N protein as it is preferred because of its relative abundance (Juniastuti et al., 2023) and is relatively stable even in variants (Mohammad et al. 2021) while very few assays are developed using the S protein as target for screening of SARS-CoV-2. As per 2022 data, among the 44 FDA-EUA antigen-detecting lateral flow assays, 42 are directed against the N antigen whereas only two target both N antigen and receptor binding domain (RBD) of the S protein (Ang et al. 2022). In one of the studies, the antigen assay developed using N protein has been able to detect all samples with high and medium-viral titers, while it could detect 64.7% (95% CI: 47.8 − 78.6%) samples in the low-virus titer cohort, but the assay is negative for PCR negatives (Funabashi et al. 2021). Even ELISA assays developed using mAbs against N protein revealed a sensitivity and specificity of 70.72% (95% CI: 66.01–75.12) and 100% (95% CI: 97.57–100), respectively, regardless of Ct values and SARS-CoV-2 variants (Yadegari et al. 2023). Another study showed a clinical sensitivity and specificity of 85.2% (95% CI, 74.3–92.0%) and 98.1% (95% CI, 93.3–99.7%) respectively against real-time PCR (Fischl et al. 2024).

Tests using commercial rapid antigen assays revealed a sensitivity ranging from 60.55 to 87.23% with high specificity ranging between 83.33 and 100% in all tests in samples with Ct value **<** 20 against the real-time PCR assay in case of Delta variants (Samsunder et al. 2023a). Similarly, while screening samples infected with Omicron sub-lineage BA.4 and BA.5, sensitivities of 73.38–74.03% and specificities of 99.22–97.41% respectively were reported using two commercial kits. Sensitivity was reported > 90% when the Ct value was < 20 (Samsunder et al. 2023b). In one of the studies where the sensitivities of rapid antigen assays were evaluated against the mutant variants, it was found that most commercially available rapid antigen tests (RATs) had similar sensitivity in detecting Omicron and Delta variants. When the antigen concentration was used as a comparator and the Ct value was used as a comparator, most rapid assays had a lower sensitivity for Omicron than Delta (Rao et al. 2023). A nanoparticle based lateral flow assay employing N protein in comparison to the RT-PCR, showed a sensitivity of 94.73% (Ding et al. 2023).

In one of the studies on performance evaluation of six commercial kits, each test showed no difference in the detection sensitivities between the wild-type virus and the variants thus suggesting that the lateral flow antigen test can be used for the detection of SARS-CoV-2 like the wild-type and the previous variants (Morinaga et al. 2023). However, some studies report regarding the reduced sensitivity of antigen tests for screening the Omicron variant in comparison with the previous variants or the wild-type virus (Osterman et al. 2022; Wagenhauser et al. 2023). As per WHO recommendations, the acceptable criteria for comparison of a rapid assay with the reference method i.e., RT-PCR needs 80% sensitivity and 97% specificity (Dinnes et al. 2022; WHO 2021), while the in-house developed assay in the present study meets these standards with 99.05% sensitivity and 100% specificity. Further, with the periodic surges of COVID-19 globally and the continuous spread of infections emphasise the importance of rapid diagnostics. In addition, there is an inherent need to make a data repository of signature motifs from these aforementioned assays which can then be established using immunoprecipitation analysis with biotinylated transcripts. These in turn could be potential targets for designing the aptamers which would ideally be specific and then test their efficacy for aptamer bound lateral flow assay towards theranostic validation.

Hence, there is the need for reliable antigen based rapid detection assays which are invaluable for the timely detection of SARS-CoV-2 infection with subsequent contact tracing and rapid isolation. However, there are some limitations to the present study, as the study population includes only symptomatic individuals. In addition, the screening of the new variant JN.1 was not done, but the developed assay may detect that too. Overall, the present study’s findings showed that the in-house developed COVID-19 rapid antigen test can exhibit excellent diagnostic performance and analytical sensitivity in detecting variants, including Omicron as the epitopes selected for S and N are derived from the regions where there are the least chances of mutations.

In response to COVID-19 infection, diagnostic real-time RT-PCR tests are adopted as a gold-standard method for detecting viral nucleic acids and identifying COVID-19 patients as the method is more sensitive than rapid antigen assays. However, the real-time RT-PCR is time-consuming, and requires equipment that cannot be used under POC settings. Antigen-based rapid assays play an invaluable role in the rapid identification of highly infectious cases, which can generally provide rapid results without the need for complex instrumentation and technical expertise.

## Data Availability

Data related to this manuscript are available with GVCVS and RP.
